# The Pathology of Rheumatism

**Published:** 1889-07

**Authors:** E. S. Pettyjohn

**Affiliations:** Chicago


### THE PATHOLOGY OF RHEUMATISM.*

By E. S. PETTYJOHN, M. D., Chicago.

Owing to the lack of definite etiological or anatomical basis for the classification of rheumatism as a disease, its study as yet is one largely of symptomatology and hypotheses.

Scudamore holds that rheumatism is a disease external to the system, which itself becomes affected in a secondary manner by sympathy, but in turn reflects back its influence upon the external parts. He says we may view the effect in the light of a

* Read before the June meeting of the Chicago Pathological Society.

common inflammation, modified by the exciting cause (cold) and by the influence of the particular textures affected.

Another theory is that the symptoms of rheumatism are set in motion by the abstraction of heat which produces contraction of the superficial vessels succeeded by dilatation and afflux of blood to the parts, dilatation being maintained long enough to result in inflammation. But the capillaries are non-muscular and the regulation of their blood supply is by nerve action.

Dr. J. K. Mitchell believed that rheumatism might have its origin in the medulla-spinalis and depend upon irritation of that organ.

Prout first suggested that the absorption of lactic acid was the cause of derangement of the secondary assimilating powers and produced rheumatism, catarrh, ague, etc., according to exposure to various influences combined with diathesis. This is also supported by Richardson, Todd, *et al.* A later view of the same theory, as promulgated by Senator, is that the acid is developed in the muscles by activity and that a chill causes a retention of this with other effete material which combined produce rheumatism. While lactic acid is found in the blood in excess in this disease, Dr. Maclagan holds that this excess is the result of increased metamorphosis of glucose (the non-nitrogenous tissue element of the motor apparatus), as a product of the effect of the rheumatic poison on the fibrous textures of the joints. He holds the miasmatic theory of rheumatism and believes the disease to be almost analagous to malaria. He charges the *bacillus malariæ* with the crime of generating both ague and rheumatism. Bertolon and Pel regard a distinct connection between rheumatism and malaria, the latter stating that in Amsterdam in the years when malaria is present there is little rheumatism.

Still another view as regards rheumatism is that it arises from a profound disturbance of the heat regulating apparatus which especially affects the muscular system, causing the heat to be generated without work, one of the consequences being that impressions of pain are conveyed to the brain by the articular nerves instead of work performed. There must be retention of heat to account for rise of temperature, but it is not heat of work done.

However much we disagree in theory we may all agree as to the clinical history of rheumatism, viz.: That it is hereditary in twenty-five per cent. of the cases; that it is more a disease of youth, especially between the ages of fifteen and thirty years; that those exposed to the weather and bad air and who are poorly nourished are especially liable to suffer from this disease; that it is prevalent in warm and temperate climates and near the sea coast; that there is greater liability, other things being equal, of its recurrence in those who have once been afflicted.

An attack is usually ushered in by a chill or rigor, followed by feverishness, stiffness or pain (which soon becomes intense) in one or more red, swollen, tender joints. This is accompanied by prostration. The patient is helpless and restless. The face is flushed; the pupils are dilated; the whole body is hot and bathed in sour-smelling perspiration. The temperature is 103 to 105 Fahr., the pulse rapid, full and soft. The tongue is covered with yellow fur; there is thirst, anorexia and constipation. The urine is diminished, dark-red and of a high specific gravity, highly acid and often contains albumen. The inflammation with all its accompaniments migrates from joint to joint, from sheath to tendon, synovial membrane to periosteum, from endocardium to neurilemma, always announcing its arrival by some or all the phenomena known of that condition. The duration is from a few days to weeks. The results are: joints stiff or anchylosed or nodulated; changes in sheaths, tendons, ligaments, periosteum, endocardium, pericardium, valves and heart-muscle, synovial membrane and neurilemma, with a medley of morbid conditions, such as hematuria, paraplegia, paralysis of the bladder from implication of the spinal cord, hemiplegia, monoplegia from cerebral disease, chorea melancholia, insanity, imbecility, etc.

A careful study of the theories just mentioned as to the cause of the group of phenomena called rheumatism will determine that each has some element of truth, supported by experimentation and clinical observation, but no one has yet gained general acceptance.

The belief that rheumatism is an infectious disease produced by bacteria is, I think, the true one, and rapidly gaining credence. Rheumatism is intimately related to other infectious diseases, especially malaria, endocarditis, meningitis and chorea, and often eventuates in neuroses. It frequently occurs in epidemics, as reported by Eichorst from the hospital at Zurich; by Pel of Amsterdam, who cites a case of a patient that was removed to a bed between two others suffering from rheumatic fever. She never having had rheumatism was attacked with it coincidently with a relapse of the disease in five other patients. Cases are mentioned by Schäfer, Salisbury and Thorensen, the latter having observed the spread of the disease by personal contact as Sawyer did malaria.

Edelfsen states that in Kiel it is a house-disease like fibrinous pneumonia and typhoid fever. He reports 728 cases occurring in 492 houses. Muller has shown that the disease does not occur within a few hours after exposure, but that it has a prodromal period as other infectious diseases, and that often the fever precedes by days the articular and other symptoms. The schizomycete or bacterium, which produces rheumatism, is taken into the blood where its action or presence may be responsible for the rapid diminution of the red corpuscles, by robbing them of their oxygen and the greater increase of the white blood corpuscles, also for the increase of the fibrin from 3 to 7 or 8 parts to 1000. The infectious organism seeks the white fibrous tissue and the serous tissues of the motor apparatus, because here it finds the best medium for growth and development. The schizomycete acts as a local irritant wherever it lodges. It alters the constituents of the blood stream, which its fills with more of its kind, and excretions are abundant. The parts inflamed produce rapid multiplication of the schizomycete and the contaminated blood carries them along to produce results elsewhere. I believe all the phenomena of this disease and its sequelæ are the result of the morbid state of the blood produced by the presence of the offending schizomycete. I am pursuaded that two or more deleterious schizomycete may exist at the same time in the system as in cases of phthisis where intercurrent attacks of rheumatism occur or where we have pneumonia in a patient with anthrax. While there are millions of microbes in the water and air and in the blood, and while the blood itself has power to diminish or even destroy by the million various bacilli in a few hours, even the typhoid bacillus as shown by Fodor; while the streptococcus pyogenes of Rosenbach is harmless in healthy saliva so long as the epithelium is intact, each of these same bacteria may produce death. Careful *post mortem* examinations have failed to find altogether adequate lesions explaining the morbid symptoms of rheumatism. Hemorrhages have been found in the mediastinum, epicardium, spleen, pleura, in the meninges and peritoneum. Also cloudy swelling of kidneys, liver and heart with injection of synovial membranes and erosion of cartilages and many morbid states producing the impression of an infectious process.

The exact schizomycete of rheumatism has not yet been found, nor are we to conclude hastily that a microbe is the cause instead of the effect of any disease. But proofs are sufficient to warrant that thorough experimental research of years, to isolate, cultivate, inoculate, and demonstrate the recurrence, the constant presence, and the positive identity of the micro-organism of rheumatism.

